# The Median Effective Concentration (EC_50_) of Epidural Ropivacaine With Different Doses of Oxycodone During Limb Surgery in Elderly Patients

**DOI:** 10.3389/fmed.2021.808850

**Published:** 2022-01-21

**Authors:** Kai Xie, Yu-long Wang, Wen-bin Teng, Rui He, Yu-hong Li, Su-qin Huang

**Affiliations:** ^1^Department of Anesthesiology, Shaoxing People's Hospital, Shaoxing, China; ^2^Department of Anesthesiology, The First Affiliated Hospital of School of Medicine, Zhejiang University, Hangzhou, China; ^3^Department of Anesthesiology, Shulan (Hangzhou) Hospital, Shulan International Medical College, Zhejiang Shuren University, Hangzhou, China

**Keywords:** EC_50_, ropivacaine, oxycodone, elderly patients, epidural

## Abstract

**Background:**

Oxycodone can be used both intravenously and epidurally in elderly patients because of its strong analgesic effect and more slight respiratory inhibition compared with other opioids at the same effect. In this study, we determined the median effective concentration (EC_50_) of epidural ropivacaine required for great saphenous vein surgery in elderly patients in order to describe its pharmacodynamic interaction with oxycodone.

**Methods:**

One hundred forty-one elderly patients scheduled for high ligation and stripping of the great saphenous vein surgery were allocated into three groups in a randomized, double-blinded manner as follows: Q2.5 group (2.5 mg oxycodone), Q5.0 group (5.0 mg oxycodone), and C group (normal saline). Anesthesia, was achieved with epidural ropivacaine and oxycodone. The EC_50_ of ropivacaine for surgery with different doses of oxycodone was adjusted by using an up-and-down sequential methods with an adjacent concentration gradient at a factor of 0.9 to inhibit analgesia. Anesthesia associated adverse events and recovery, characteristics were also recorded.

**Results:**

The EC_50_ of ropivacaine for the great saphenous vein surgery in elderly patients was 0.399% (95% CI, 0.371–0.430%) in the Q2.5 group, 0.396% (95% CI, 0.355–0.441%) in the Q5.0 group, and 0.487% (95% CI, 0.510–0.465%) in the C group, respectively (*P* < 0.05). Specially, the EC_50_ of ropivacaine in the Q2.5 and Q5.0 groups was lower than that in the C group (*P* < 0.01), But the difference between the Q2.5 group and the Q5.0 group was not significant (*P* > 0.05). There was no significant difference in the Bromage score from the motor block examination, heart rate (HR) or mean arterial pressure (MAP) at each observation time point after epidural administration among the three groups (*P* > 0.05). No serious adverse reactions occurred in any of the three groups.

**Conclusion:**

Oxycodone combined with ropivacaine epidural anesthesia can reduce the EC_50_ of ropivacaine required for elderly patients undergoing the great saphenous vein surgery. There was no significant difference in anesthesia associated adverse events among the three groups. The recommended dose of oxycodone is 2.5 mg.

## Background

Varicosity of the great saphenous vein is more common in patients who have long been engaged in standing work, especially in the elderly. High ligation and stripping of the great saphenous vein under epidural anesthesia is a classic operation for the treatment of the disease (1). With the gradual reduction in the size and function of their organs, elderly individuals often suffer from hypertension, diabetes, coronary heart disease, and other medical diseases. High-dose or high-concentration epidural injection of local anesthetics might cause hypotension, bradycardia, nausea, anxiety, and other adverse reactions, which may induce cardiovascular events and central nervous system toxicity in elderly patients or patients with cardiovascular diseases (2). In addition, the block level of local anesthetics in elderly patients is much higher than that in younger patients and is often accompanied by motor block (3). To reduce the risks from epidural anesthesia in elderly patients, it is necessary to reduce the concentration of the local anesthetics, but too low a concentration may lead to an insufficient postoperative analgesic effect.

Previous studies have reported that the epidural combinations involving low-dose opioids can reduce the concentration of local anesthetics, ensure sufficient depth of anesthesia, and reduce the incidence of adverse reactions associated with systemic administration (4). Morphine has been the most frequently used opioid, which provides effective analgesia but is associated the possibility of inducing adverse effects such as nausea, vomiting, pruritus, and respiratory depression, which limits its use (5). Our team's previous study have shown that addition of sufentanil can reduce the median effective concentration (EC_50_) of ropivacaine (6). Oxycodone is another kind of drug with strong analgesic effect and less adverse reactions. At present, it has been widely used in intraoperative (5) and postoperative analgesia (7). It is also be safely and effectively used in epidural anesthesia in elderly patients (8) with few studies regarding its effect on the EC_50_ of epidural ropivacaine for elderly patients. Therefore, the aim of this study was to assess oxycodone on the EC_50_ of ropivacaine in elderly patients undergoing epidural anesthesia. In this study, Dixon's up-and-down sequential allocation was used to explore the EC_50_ of ropivacaine when combined with oxycodone in the epidural anesthesia of elderly patients undergoing high ligation and stripping of the great saphenous vein to provide a reference for the clinical medication of elderly patients.

## Methods

### Subject Selection

Elderly patients undergoing high ligation and stripping of the great saphenous vein under elective epidural anesthesia in Shaoxing People's Hospital from April 2018 to April 2019 were initially enrolled. All patients were aged from 60 to 86 years, with a body mass index (BMI) <30 kg/m^2^ and American Society Anesthesiologists (ASA) physical status grade I or II. Patients with hypotension, low blood volume, puncture site infection, bacteremia; thrombocytopenia, prolonged clotting time, coagulation dysfunction, spinal deformity or surgery, or suffering from Alzheimer's disease were excluded. This study was discussed and approved by the ethics committee of Shaoxing People's Hospital (No: 201801) and registered as a clinical trial (ChiCTR 180015025). All patients signed an informed consent form.

### Study Design and Grouping

This study was a randomized, double-blind, prospective study. The patients were randomly divided into three groups by computer, and the drugs were prepared by a designated person. The anesthesiologists, who were not aware of the medication plan performed the puncture operation. The test data were observed, evaluated, and recorded. When severe adverse reactions occurred such as severe allergic reaction or total spinal anesthesia, the patient was unblinded midway and excluded. Other patients were finally unblinded after statistics. A total of 141 patients were randomly divided into three groups: Q2.5 group: ropivacaine combined with low-dose oxycodone group; Q5.0 group: ropivacaine combined with high-dose oxycodone group; and the C group: ropivacaine alone.

### Preoperative Preparations and Anesthesia Protocol

No patients had taken their medication before entering the room. After the patient entered the room, upper extremity venous access was obtained, and Ringer's solution (500 ml) was infused. Echocardiography (ECG), heart rate (HR), SpO_2_, and non-invasive blood pressure were routinely monitored. The patient was placed in the left lateral position for epidural puncture at the L_1−2_ level. When the needle reached the epidural space and, no blood or cerebrospinal fluid was found by slow suction, the puncture was considered successful after exclusion into the blood vessel or subarachnoid space. A catheter was placed 3 cm head-on and fixed properly. The patient was then laid on his or her back and, injected with 2% weight/volume lidocaine 2 ml, and no adverse reaction was confirmed 5 min later. According to the results of previous studies (6, 9) and pretests, local anesthetics were injected slowly into the epidural space at a dose of 15 ml (with or without oxycodone) over 1 min. The Q2.5 group and the Q5.0 group were given 2.5 and 5 mg oxycodone, respectively, while the C group was given none. According to the results of the pretest, the concentration of ropivacaine in the first patient of C group was set at 0.51% weight/volume. The concentration of ropivacaine in the first patients of the Q2.5 group and the Q5.0 group was 0.46% weight/volume; after administration, the patients were immediately placed supine for observation, and the sensory and motor block were tested every 5 min. According to the sequential method, the concentration of ropivacaine in the next patient was adjusted according to the reaction of the previous patient: if the bilateral pain block in the L_1_-L_5_ plane of the operation area was effective within 30 min after epidural administration, the concentration of ropivacaine of the next patient was decreased by 1 gradient (multiplication of the current patient's dose by 0.9); if the bilateral pain in the L_1_-L_5_ plane of the operation area is not completely blocked within 30 min after epidural administration, the concentration of ropivacaine in the next patient was increased by 1 gradient (division of the current patient's dose by 0.9); if puncture failed, the blood vessels were punctured, the catheter failed or unilateral anesthesia or no anesthesia, effect occurred within 30 min after epidural administration, the concentration of ropivacaine in the next patient was the same as that of the current patients. In cases of ineffective epidural anesthesia or puncture failure, the anesthesiologist in charge should decide to increase the dose of local anesthetics or change to other anesthesia methods. After epidural administration, the patient was observed while lying on his or her back. If SpO_2_ was lower than 95% under spontaneous breathing, the patient was given an oxygen mask; if hypotension occurred (more than 30% lower than preoperative blood pressure), ephedrine was given to restore blood pressure; if bradycardia occurred (HR < 50 bpm), atropine 0.5 mg was injected intravenously; if nausea and vomiting occurred, symptomatic treatment was given.

### Measurements

General patient information was recorded. Mean arterial pressure (MAP), HR, SpO_2_, and sensory and motor block test results were recorded every 5 min after epidural administration. According to the Hollman grading method (10), the sensory block was evaluated as follows: grade 0 = sensitive to acupuncture pain; grade 1 = insensitive to acupuncture pain; grade 2 = no pain to acupuncture; and grade 3 = no sense to acupuncture. According to the modified Bromage method (11), the motor block was evaluated as follows: 4 = the patient can move the knee and foot; 3 = the patient can move the knee freely; 2 = the patient cannot bend the knees, but the feet can move; and 1 = knees and feet cannot move. The onset time of the sensory block (L_1_-L_5_ plane acupuncture test reached Hollman grade 2–3 after epidural administration), the highest cephalic plane of sensory block and the occurrence of adverse reactions were recorded. The simulated pain visual score was recorded using a visual analog scale (VAS) every 30 min within 2 h after surgery, every 1 h from 2 to 12 h after surgery, and every 4 h from 12 to 48 h after surgery. A score of 0, it indicated that the patient did not feel pain and was in a quiet state; a score between 1 and 3 indicated the feeling of slight but tolerable pain after the operation, a score between 4 and 6 indicated that the pain after operation has reached a medium level and can be endured but with occasional groaning, requiring medical treatment; a score of 7–10 points indicated severe completely intolerable pain requiring drug analgesia. The postoperative hospital stay was also recorded.

### Sample Size Determination

Sample size was estimated based on assuming a standard deviation (SD) of 0.35 of the mean. Power was given at 0.80 to detect a 15% difference in the mean between groups at *P* < 0.05 with use of G^*^Power 3.0.10. Therefore, a total of 135 patients was calculated at the minimum sample size. To prevent the effects of dropout, two patients in each group were added, for a total of 141 patients.

### Statistical Analysis

SPSS 11.0 software (SPSS, USA) and GraphPad Prism7.0 software (version, USA) were used for statistical analysis. Normally distributed data are expressed as the mean (SD). One way ANOVA was used for comparisons between groups, and the Dunnett T3 test was used for *post-hoc* pairwise comparisons. Date with a skewed distribution were expressed as the median (interquartile interval, Q1~Q3). Comparison between groups were performed by kruskal-wallis by ranks and then by the Tukey's multiple comparisons test; counting data were compared by the χ^2^-test. The test level (α) was set to 0.05. The EC_50_ and 95% confidence interval (95% CI) of ropivacaine for anesthesia during high ligation and stripping of the great saphenous vein in elderly patients were calculated by the sequential method (12).

## Results

### General Information

**Figure 1:**
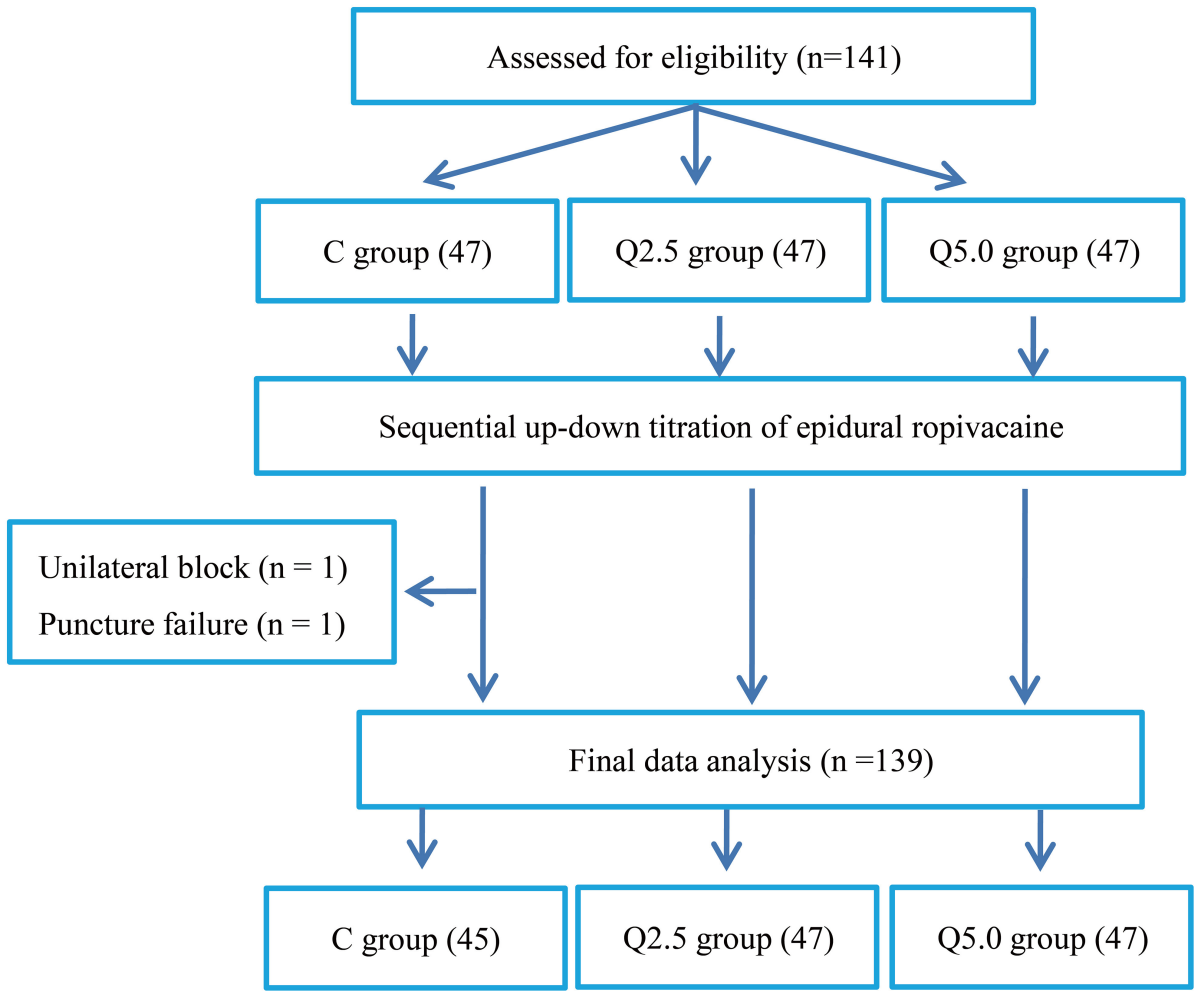
Flow diagram of the study.

A total of 141 subjects were included in this study, 47 in each group. Two patients in the C group were withdrawn from the trial due to unilateral block (1 patient) and puncture failure (1 patient), and 139 patients finally completed the trial (Figure 1). Table 1 shows that there were no significant differences in sex, age, height, BMI, operation time, Bromage score, or the highest level of analgesia block among the three groups (*P* > 0.05). The onset time of analgesia in the Q2.5 group was faster than that in the C group (*P* < 0.05) but the same as that in the Q5.0 group.

**Table 1:** Demographic data and patients' characters.

	**C group**	**Q2.5 group**	**Q5.0 group**
	**(*n* = 45)**	**(*n* = 47)**	**(*n* = 47)**
Sex (M/F)	27/18	31/16	33/14
Age (year)	67 (6)	67 (4)	67 (6)
Weight (kg)	66 (10)	66 (9)	66 (9)
ASA physical status class (I/II)	30/15	32/15	34/13
Height (cm)	165 (7)	165 (7)	167 (7)
BMI (kg·m^−2^)	24 (3)	24 (2)	24 (3)
Onset time of analgesia (min)	13 (12~14)	11 (10~12)^*^	12 (10~14)
Highest level of block	T_8_ (T_8_~T_10_)	T_8_ (T_6_~T_10_)	T_9_ (T_8_~T_10_)
Operation time (min)	54 (14)	57 (13)	57 (16)
Bromage score	4 (4~4)	4 (4~4)	4 (4~4)

*Data are expressed as the mean (SD), number of patients or median (Q_1_~Q_3_); C group: ropivacaine alone; Q2.5 group: ropivacaine combined with low-dose (2.5 mg) oxycodone; Q5.0 group: ropivacaine combined with high-dose (5.0 mg) oxycodone. Tukey's multiple comparisons test*:

* *P < 0.05, compared with the C group*.

### Hemodynamics

**Figure 2:**
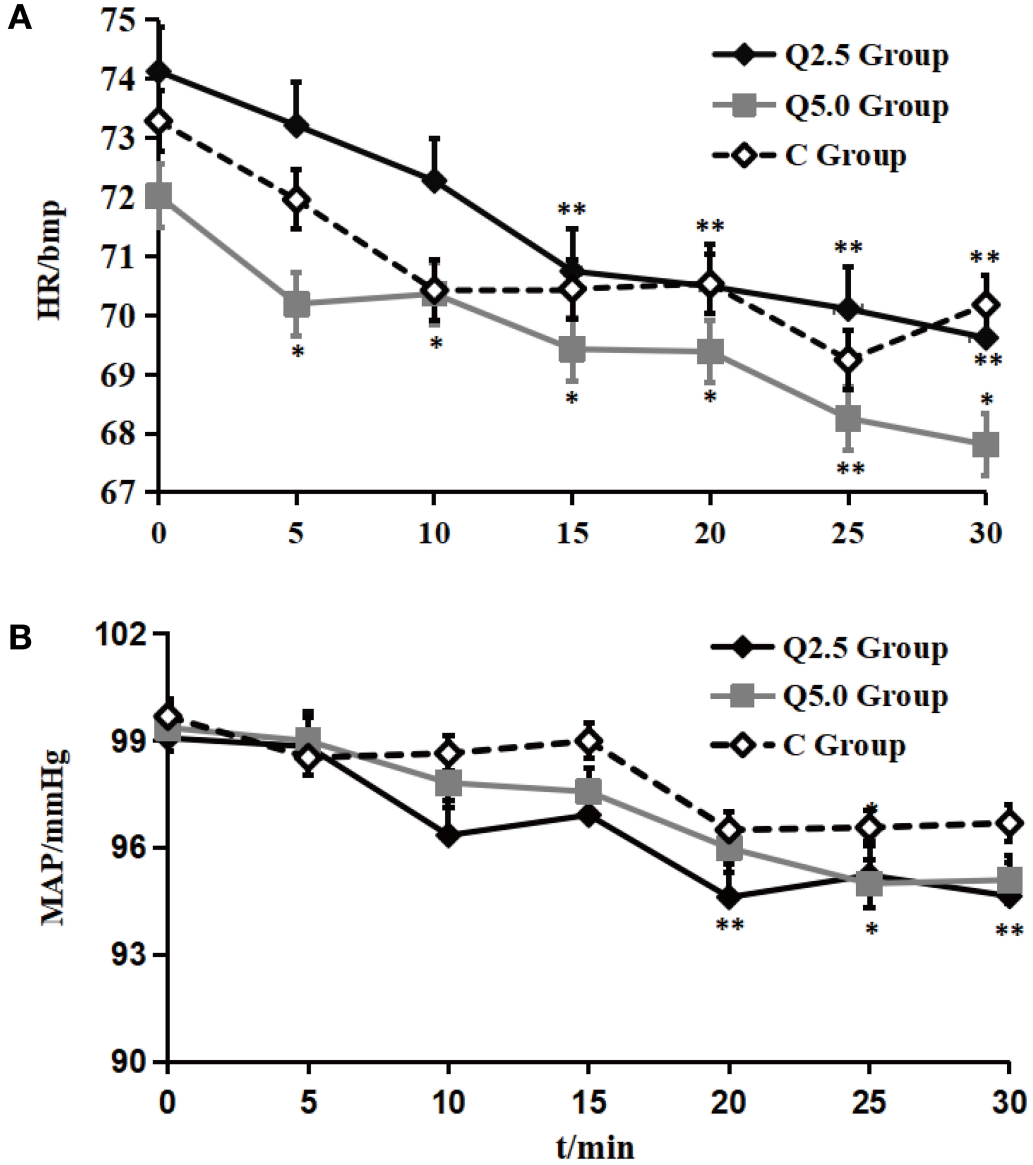
Hemodynamic changes before (0 min) and after epidural injection. Data are expressed as the mean (SD); C group: ropivacaine alone, Q2.5 group: ropivacaine combined with low-dose (2.5 mg) oxycodone; Q5.0 group: ropivacaine combined with high-dose (5.0 mg) oxycodone; **(A)** Changes in HR; **(B)** Changes in MAP; Dunnett T3 test: *P < 0.05, **P < 0.01, compared with the baseline.

Because of preoperative fasting, the vascular volume of patients undergoing elective surgery is relatively deficient. After epidural anesthesia, the sympathetic nerve is inhibited, and blood vessels are dilated. Compared with those before anesthesia administration, HR and MAP decreased in the three groups, but there was no significant difference among the three groups (*P* > 0.05). The data are shown in Figure 2.

### Median Effective Concentration

**Figure 3:**
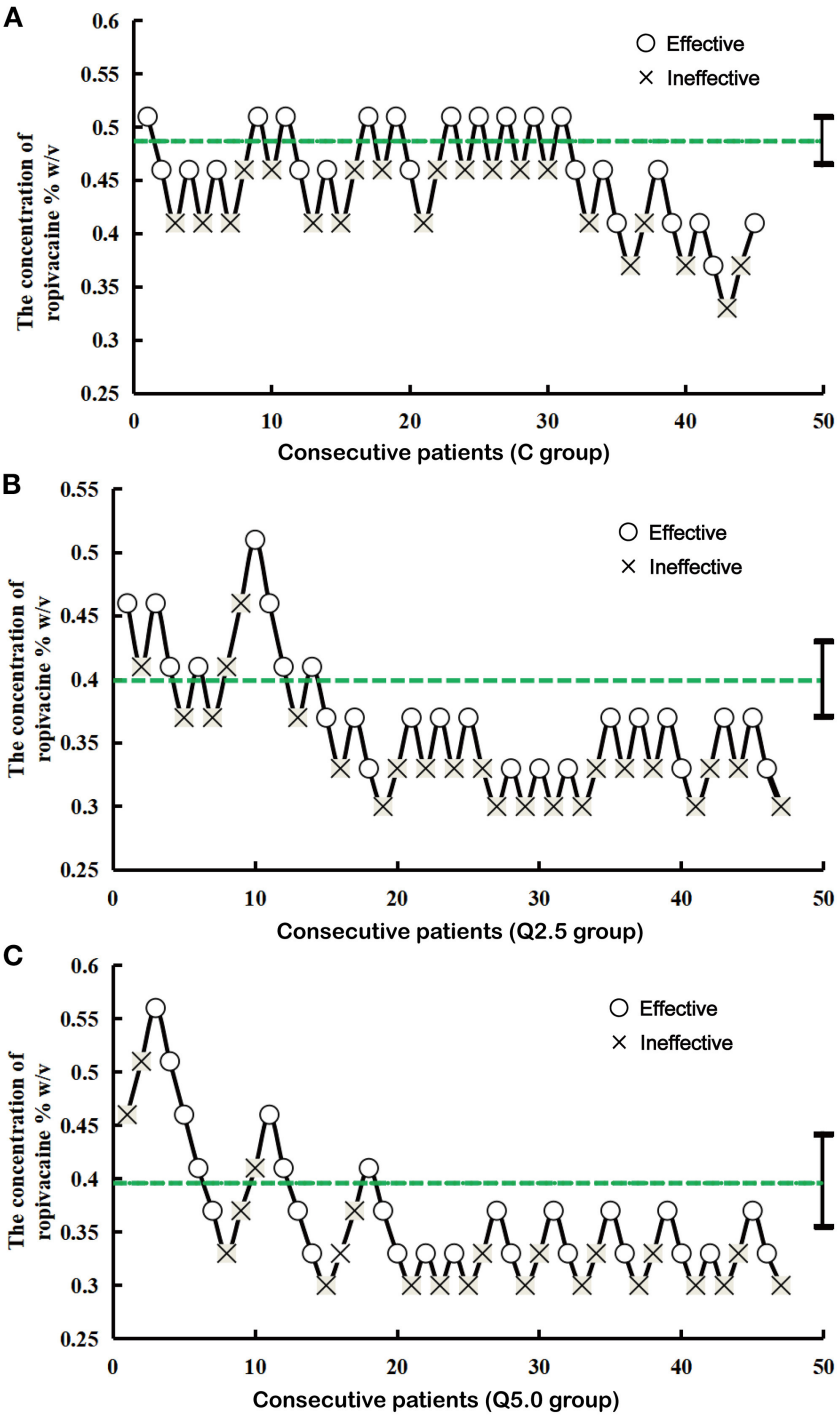
Consecutive ropivacaine concentration during great saphenous vein surgery for determining EC_50_. The lines represent the mean ropivacaine (%, w/v) concentration when crossing from a failure to inhibit analgesia. The average of these concentrations is defined as the EC_50_. The EC_50_ of ropivacaine was 0.487% with a 95% CI of 0.510–0.465% in the C group **(A)**, 0.399% with a 95% CI of 0.371–0.430% in the Q2.5 group **(B)**, and 0.396% with a 95% CI of 0.355–0.441% in the Q5.0 group **(C)**.

As shown in Figure 3 the EC_50_ of ropivacaine required to achieve epidural block was 0.399% (95% CI, 0.371–0.430%) in the Q2.5 group, 0.396% (95% CI, 0.355–0.441%) in the Q5.0 group, and 0.487% (95% CI, 0.510–0.465%) in the C group. The EC_50_ of the C group was significantly higher than that of the Q2.5 group and the Q5.0 group (*P* < 0.01), but there was no significant difference between the values for the Q2.5 group and the Q5.0 group (*P* > 0.05).

### Adverse Reactions

There were no serious adverse reactions in the three groups. In the C groups, hypotension occurred in two patients, and the blood pressure returned to normal after using ephedrine; and vomiting occurred in one patient, which improved spontaneously. In the Q2.5 group, bradycardia occurred in one patient, and the HR returned to normal after using atropine; and dizziness occurred in one patient, which improved after oxygen inhalation. Two cases of hypotension occurred in the Q5.0 group, and the blood pressure returned to normal after intravenous ephedrine.

The VAS pain score in the Q5.0 group 2 h after surgery was slightly lower than that in the other two groups, but there was no significant difference among the three groups (*P* > 0.05); There were six patients with VAS scores >4 in the Q2.5 group, five in the Q 5.0 group, and four in the C group. The VAS pain score >4 in group Q 5.0 occurred earlier than in the other two groups (*P* < 0.01). There was no significant difference in postoperative hospital stay (*P* = 0.149) among the three groups (Table 2).

**Table 2:** Comparison of postoperative pain 2 h after surgery and postoperative hospitalization days among the three groups.

**Groups**	**VAS and time**			**Hospital stay (d)**
	**VAS score at 2 h postop**	**Time to VAS score >4 postop (h)**	**Incidence of VAS score >4**	
Q2.5 group	3 (2~3)	9 (7~11)^**^	12.77%	4 (3~4)
Q5.0 group	2 (2~3)	2 (2~2)	10.64%	4 (4~5)
C group	3 (3~3)	8 (6~10)^**^	8.89%	4 (4~6)
F/χ^2^	4.154	8.547	0.283	3.815
P	0.125	0.006	0.868	0.149

*Data are median (Q_1_~Q_3_); C group: ropivacaine alone group; Q2.5 group: ropivacaine combined with low-dose (2.5 mg) oxycodone; Q5.0 group: ropivacaine combined with high-dose (5.0 mg) oxycodone*;

** *P < 0.01, compared with Q5.0 group*.

## Discussion

In this study, the EC_50_ of ropivacaine was determined by the sequential method. The advantage of the sequential method is that it can reduce the number of samples and improve the test efficiency under the same sample size (13, 14). Generally, 20–40 patients were required by the sequential method (15, 16). To prevent the elimination of patients during the trial, the number in each group was set to 47. According to the pretest, the initial concentration of ropivacaine was 0.51% in the single drug group and 0.46% in the combination group. The results showed that the EC_50_ of ropivacaine for epidural anesthesia in the C group, the Q2.5 group and the Q5.0 group were 0.487, 0.399, and 0.396%, respectively. The EC_50_ of ropivacaine in the C group was higher than that in the Q2.5 group and the Q5.0 group, while that in latter two groups was the same.

Local anesthetics exert analgesic effects by inhibiting sodium channels, while oxycodone exerts analgesic effects by exciting opioid receptors. The two drugs achieve analgesic effects through different mechanisms (17). High doses or high concentrations of epidural ropivacaine can cause hemodynamic fluctuations in elderly patients, especially a decrease in blood pressure (18). In this study, the HR and MAP of the three groups all decreased after treatment, including one case of bradycardia in the Q5.0 group and two cases of hypotension in the C group, but there was no significant difference in hemodynamic changes among the three groups. This may be related to the fact that the patients included in this study were ASA physical status class I or II, there was no reports of cardiovascular disease before the operation, and the dose of ropivacaine was low. The effect of oxycodone combined with local anesthetics on the hemodynamics of patients with cardiovascular dysfunction needs further study.

Previous studies have shown that opioids combined with local anesthetics can reduce the dosage of the latter (6). The same dose of epidural oxycodone produces a better analgesia effect than intravenous administration; to achieve the same analgesic effect, the dose required for epidural administration would be significantly lower than that for intravenous administration (19), indicating that the epidural application of oxycodone combined with local anesthetics has certain advantages. Studies have shown that oxycodone combined with local anesthetics can accelerate the onset time of analgesia, significantly prolong the analgesic time and enhance the effect of epidural analgesia (8, 20). Based on the above studies, this study chose to combined oxycodone 2.5 and 5.0 mg with ropivacaine. The present results showed that compared with the single drug group, the dose of ropivacaine in the combined drug group was reduced by 22%, and the onset time of analgesia was 1–2 min faster, which is consistent with the previous studies. The EC_50_ of ropivacaine in the high-dose and low-dose oxycodone groups was similar, probably because oxycodone 2.5 mg reaches the top of the S-shaped curve, and thus the analgesic effect no longer increases with increasing of dose.

There are also some limitations in this study. Firstly, based on previous researches (8, 21), a single injection of 15 ml local anesthetics was used in the present study. As far as I know, after excluding the accidental entry of epidural catheter into subarachnoid space and blood vessels, a single injection is usually used for epidural anesthesia, in order to shorten the onset time and increase the block level of local anesthetics. In any case, this is not a generally recommended clinical procedure and may have a certain risk. Therefore, it is this limitation that does reduce the clinical utility of the present study in terms of recommendations. In the future studies, local anesthetics will be given based on the medication guidelines. Secondly, there are many factors that influence the anesthetic effect. In addition to drug interactions, the influence of genetic variation on drug effects should also be considered (22, 23). The influence of gene polymorphisms on the drug effect of oxycodone also needs further study.

There was no difference in the pain scores among the three groups 2 h postoperatively, and the time to achieve a VAS score of more than four postoperatively was shorter in the Q5.0 group than in the Q2.5 and C group, which may be related to the lower concentration of ropivacaine in the Q5.0 group than in C group, suggesting that postoperative analgesics should be added for patients under lower concentrations of epidural ropivacaine anelgesia. Olczak et al. (8) found that oxycodone at a dose of 5 mg administered epidurally combined with 15 ml 0.25% bupivacaine and sedation with propofol infusion at a dose of 3–5 mg/kg/h prolonged the analgesia speriod to ~10 h in patients after total hip arthroplasty. The main adverse reactions epidural oxycodone administration are pruritus, nausea and vomiting, mild respiratory depression, and so on (24); however, our experimental design is different from that of the above study. The inclusion of oxycodone, whether epidural or intravenous, can increase the analgesic effect but also, potentially, the adverse reactions of oxycodone. In this study, adverse reactions occurred in four patients in the entire cohort, including bradycardia, dizziness, and hypotension, which was similar to that in the single drug group. The inclusion of oxycodone did not lead to an increase in adverse reactions. No respiratory depression or postoperative pruritus caused by epidural oxycodone was found in this study.

## Conclusions

Oxycodone combined with ropivacaine epidural anesthesia for elderly patients undergoing high ligation and stripping of the great saphenous vein can reduce the EC_50_ of ropivacaine and shorten the onset time of anesthesia. The recommended dose of oxycodone is 2.5 mg.

## Data Availability Statement

The raw data supporting the conclusions of this article will be made available by the authors, without undue reservation.

## Ethics Statement

The studies involving human participants were reviewed and approved by the Ethics Committee of Shaoxing People's Hospital. The patients/participants provided their written informed consent to participate in this study.

## Author Contributions

KX and Y-lW: conception, design of the research, and writing of the manuscript. KX: acquisition of data. W-bT: analysis and interpretation of the data. RH, Y-hL, and W-bT: statistical analysis. Y-hL: obtaining financing. Y-hL and S-qH: critical revision of the manuscript for intellectual content. All authors contributed to the article and approved the submitted version.

## Funding

This study was supported by Zhejiang Provincial Department of Science and Technology Fund (Grant Nos. LGF19H030011 and LY21H150001), Zhejiang Provincial Health Committee Fund (Grant No. 2020KY329), Shaoxing Science and Technology Bureau Fund (Grant No. 2020A13014), Key Fund of Shaoxing People's Hospital (Grant No. 2020YA05), and Shaoxing Key Discipline of Anesthesiology (Grant No. 2019szd04).

## Conflict of Interest

The authors declare that the research was conducted in the absence of any commercial or financial relationships that could be construed as a potential conflict of interest. The reviewer LZ declared a shared affiliation with several of the authors, KX, Y-lW, and RH, to the handling editor at the time of review.

## Publisher's Note

All claims expressed in this article are solely those of the authors and do not necessarily represent those of their affiliated organizations, or those of the publisher, the editors and the reviewers. Any product that may be evaluated in this article, or claim that may be made by its manufacturer, is not guaranteed or endorsed by the publisher.
